# *PIK3CA* mutations in breast cancer: A Tunisian series

**DOI:** 10.1371/journal.pone.0285413

**Published:** 2023-05-17

**Authors:** Mariem Ben Rekaya, Farah Sassi, Essya Saied, Linda Bel Haj Kacem, Nada Mansouri, Sinda Zarrouk, Saifeddine Azouz, Soumaya Rammeh

**Affiliations:** 1 Faculty of Medicine of Tunis, UR17ES15, University Tunis El Manar, Tunis, Tunisia; 2 Pathology Department, Charles Nicolle Hospital, Tunis, Tunisia; 3 Pathology Department, Military Hospital, Tunis, Tunisia; 4 Pasteur Institute of Tunis, Genomics Platform, University of Tunis El Manar Tunis, Tunisia; King Faisal Specialist Hospital and Research Center, SAUDI ARABIA

## Abstract

**Background:**

The aim of this study was to analyze *PIK3CA* mutations in exons 9 and 20 in breast cancers (BCs) and their association with clinicopathological characteristics.

**Methods:**

Mutational analysis of *PIK3CA* exon 9 and 20 was performed by Sanger sequencing in 54 primary BCs of Tunisian women. The associations of *PIK3CA* mutations with clinicopathological characteristics were analyzed.

**Results:**

Fifteen exon 9 and exon 20 *PIK3CA* variants were identified in 33/54 cases (61%). *PIK3CA* mutations including pathogenic (class 5/Tier I) or likely pathogenic (class 4/Tier II) occurred in 24/54 cases (44%): 17/24 cases (71%) in exon 9, 5/24 cases (21%) in exon 20 and 2/24 cases (8%) in both exons. Of these 24 cases, 18 (75%) carried at least one of the three hot spot mutations: E545K (in 8 cases), H1047R (in 4 cases), E542K (in 3 cases), E545K/E542K (in one case), E545K/H1047R (in one case) and P539R/H1047R (in one case). Pathogenic *PIK3CA* mutations were associated with negative lymph node status (p = 0.027). Age distribution, histological SBR tumor grading, estrogen and progesterone receptors, human epidermal growth factor receptor 2, and molecular classification were not correlated with *PIK3CA* mutations (p > 0.05).

**Conclusion:**

The frequency of somatic *PIK3CA* mutations in BCs of Tunisian women is slightly higher than that of BCs of Caucasian women and more observed in exon 9 than in exon 20. *PIK3CA* mutated status is associated with negative lymph node status. These data need to be confirmed in larger series.

## Introduction

Breakthroughs in molecular biology have clearly established that breast cancer (BC) is a cell signaling disease. Phosphatidylinositol 3-kinase/lipid kinase B/mammalian target of the rapamycin (PI3K/AKT/mTOR) pathway is one of the major deregulated pathways in human cancers, especially in BC [1,2].

The p110α catalytic subunit (PI3K p110α) oncogene encoded by *PIK3CA* is a lipid kinase which regulates cell proliferation, catabolism, cell adhesion and apoptosis. Single base and insertions/deletions (indels) are the most frequent *PIK3CA* alterations observed in 13% of solid tumors [3]. However, the frequency of PIK3CA mutations differs among populations and varies among cancer types, stages and ethnicity. The role of ethnicity in frequency rate disparity of *PIK3CA* mutations has been showed in head and neck squamous cell carcinomas [4,5].

*PIK3CA* gene is mutated in 18–45% of BCs [5], with over 80% of mutations clustering within three hot spots: two of the helical domain (exon 9, commonly E542 and E545) and one of the kinase domain (exon 20, commonly H1047) [5,6].

Two-third of BCs express estrogen and progesterone receptors (ER/PR) and lack human epidermal growth factor receptor 2 (HER2) overexpression, for which endocrine therapy is the primary drug option. However, approximately 30% of BC patients carry mutations in the *PIK3CA* gene, which are associated with resistance to endocrine therapy [7]. This is due to multiple mechanisms including dysregulated PI3K/AKT/mTOR signaling [8]. With the emergence of PI3K inhibitors, it is important to identify patients who may benefit from this therapy [8]. Alpelisib is an oral alpha-specific PI3K inhibitor administred in combination with fulvestrant for the treatment of postmenopausal women with hormone receptors positive and HER2 negative, *PIK3CA*-mutated, advanced or metastatic BC with progression after endocrine therapy [9]. Conflicting correlations between *PIK3CA* mutations and clinicopathological data have been reported [10]. In early-stage disease, PIK3CA mutations are significantly associated with better invasive disease-free, distant disease-free, and overall survivals [11].

To our knowledge, this study is the first to analyze *PIK3CA* mutations in exon 9 and exon 20 in BCs of Tunisian patients.

## Material and methods

### Patients, tissue samples, DNA extraction and PCR reaction

Fifty-four Formalin-Fixed Paraffin-Embedded (FFPE) primitive BC specimens were selected from the pathology departments of Military and Charles Nicolle hospitals (Tunis). All the samples were obtained from women who did not receive preoperative treatment. The diagnosis of BC was made on core biopsies in 7 cases (13%), lumpectomies in 15 cases (28%) and mastectomies in 32 cases (59%).

Clinicopathological data were obtained from pathology records. A pathologist reviewed slides and selected areas rich in tumor cells (at least 20%) avoiding poorly fixed and necrotic areas. Selected areas from FFPE tissues of the 47 surgical specimens (lumpectomy/mastectomy) were manually macrodissected using a mechanical punch and were recuperated in sterile Eppendorf tubes. For core biopsies (n = 7), 4 or 5 FFPE sections (5-6μm thickness) were obtained. Blocks of each case were cut with a new blade to avoid carry-over contamination and the bloc holder and the plate of the microtome were disinfected. Only tissue enriched on tumor cells have been collected without healthy tissue or blood samples.

The total DNA was extracted using QIAamp FFPE kit (Qiagen, Germany) according to the manufacturer’s instructions. The nucleic acid concentration and DNA purity were measured using a NanoDrop 1000 (Thermofisher Scientific, Waltman, MA, USA). The double strand DNA was measured by the Denovix fluorometer with dsDNA Broad Range Assay having a standard detection range from 2 to 2000 ng total mass in 200 μl volumes.

Primer pair have been designed using the Primer 3 version 4 software to amplify exon 20 and exon 9 of *PIK3CA* gene, avoiding the frequent cross-amplification of chromosome 22q (a known *PIK3CA* pseudogene). Primer sequences, annealing temperatures (Ta) and product lengths are listed in Table 1. A final concentration of 0.4 μM of each primer and 20 to 50 ng of template DNA were used per reaction. Amplification conditions involved a heat-activation step of 15 min at 95°C, followed by 35 cycles of 94°C for 30 seconds, 55°C for 30 seconds and 72°C for 30 seconds followed by a final extension step at 72°C for 30 min to perform entire elongation of all neosynthesized DNA strands. The amplified fragments were visualized in 2% agarose gel stained with EasyStain I (Biomatik).

**Table 1:** Primer sequences, annealing temperatures and product lengths.

Exon	Primername	Primer sequence 5’→3’	Tm (°C)	Ta (°C)	Amplicon length (bp)
9	PIK9F	GGGAAAAATATGACAAAGAAAGC	58	55	192
	PIK9R	CCATTTTAGCACTTACCTGTGAC	58		
20-I	PIK20F1	CATTTGCTCCAAACTGACCA	59		245
	PIK20R1	TGTGCATCATTCATTTGTTTCA	60		
20-II	PIK20F2	TTGATGACATTGCATACATTCG	59		236
	PIK20R2	GGTCTTTGCCTGCTGAGAGT	59		

Tm: Melting Temperature; Ta: Annealing Temperature; bp: Base pairs.

### Ethical considerations

Ethical approval was obtained from the medical Ethics Committee of Charles Nicolle Hospital of Tunis. All patients gave written informed consent for publication of clinical and laboratory data. Patients were fully anonymized.

### Sanger sequencing and variant analysis

To remove unused dNTPs and primers, PCR products were purified using the enzymatic method Exo-SAP PCR Product Cleanup Reagent. PCR sequencing has been performed using the Big Dye Terminator Kit V.3.1 (Applied Biosystems, Foster City, CA, USA). To remove both unlabeled and labeled dye, sequencing reactions were purified using BigDye Xterminator Purification (Life Technologies). Sequences analysis was performed in the Applied Biosystem 3500 Genetic Analyzer. Sequence reading was performed using the BioEdit sequence alignment editor. Variants including those with unknown function were annotated using Mutation Taster, Polyphen and SIFT tools and Sequence Variant Nomenclature was performed according to the guidelines of the Human Genome Variation Society (HGVS) using the Mutalyzer program and the reference sequence NM_006218.4. Free variants databases: dbSNP, ClinVar, ClinGen, COSMIC, and the Clinical Knowledge base were queried to identify known variants and published data on clinical significance and to collect the reference identifier (rs) and the cosmic ID of each variant. Alamut Plus tool (from SOPHiA GENETICS) has been used to check annotation and function prediction. Variants classification has been performed according to ACMG/AMP guidelines for somatic sequence variant interpretation [12].

### Immunohistochemistry

Data including ER, PR and HER2 immunohistochemical expression were obtained from pathology records. Molecular subtypes were defined based on the St Gallen’s criteria [13]. Chromogenic *In Situ* Hybridization (CISH) was performed for HER2 score 2+. All HER2 test results were classified according to 2018 ASCO/CAP HER2 testing recommendations [14].

### Statistical analysis

Statistical analyses were performed using SPSS 26.0. The χ2 and Fisher’s tests were used to determine associations between pathogenic and likely pathogenic mutations (class 5/Tier I and class 4/Tier II) of *PIK3CA* gene and clinicopathological features of BCs (Age, lymph node status, ER, PR, HER2 and molecular classification). P-values <0.05 were considered significant.

## Results

### Clinicopathological characteristics of cases

The mean age of patients was 51 years (31–84). Median tumor size was 3 cm (0.8cm-10 cm). Invasive ductal carcinoma was diagnosed in 50/54 cases (93%) and invasive lobular carcinoma in the remaining 4 cases (7%). Based on the SBR grading system, 4 cases (8%) were grade I, 25 (46%) were grade II, and 25 (46%) were grade III. Lymph node status was known for 45 cases, with 29 cases (64%) having metastatic BC. Immunohistochemically, 38 cases (70%) were ER positive, 36 (67%) were PR positive, and 4 cases (7%) showed overexpression of HER2. BCs were classified as luminal A in 40 cases (74%), luminal B in 5 cases (9%), HER2+ in 4 cases (8%), and triple negative in the remaining 5 cases (9%).

### Mutational analysis

**Fig 1:**
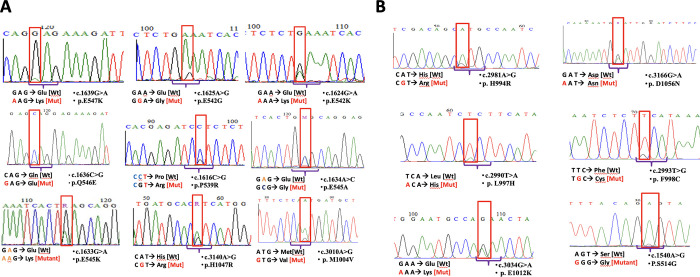
Electropherograms of the 15 *PIK3CA* variants identified in breast cancers (A) Known *PIK3CA* mutations (B) Novel variants. All sequences are in sense strand. Red rectangle box shows the position of the mutations. The major peak corresponds to the normal nucleotide and the minor peak corresponds to the mutant nucleotide. Altered nucleotide and amino acid positions and related codon substitution are shown of each corresponding sequence.

**Fig 2:**
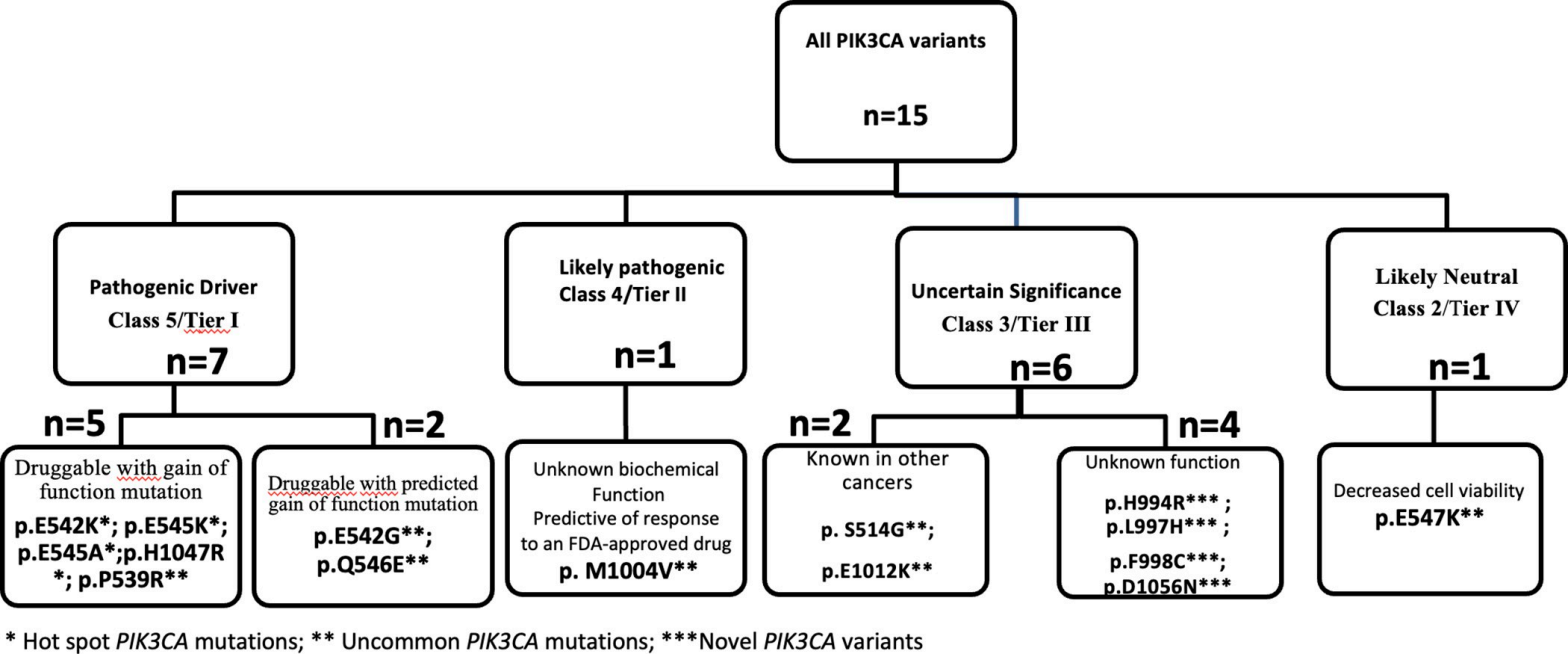
*PIK3CA* variant’s classification according to the American College of Medical Genetics and Genomics guidelines.

Fifteen exon 9 and exon 20 *PIK3CA* variants were identified in 33 cases (61%): 24/54 (44%) had pathogenic mutations classified as class 5 or 4 and 9/54 (17%) had mutations with uncertain significance (Figs 1 and 2).

Pathogenic mutations were observed in 17/24 cases (71%) in exon 9, in 5/24 cases (21%) in exon 20 and in 2/24 cases (8%) in both exons. Of these cases, 18 (75%) carried at least one of the three hot spot mutations: E545K (in 8 cases), H1047R (in 4 cases), E542K (in 3 cases), E545K/E542K (in one case), E545K/H1047R (in one case) and P539R/H1047R (in one case). H1047L was not found. *PIK3CA* mutations were class 3 in 10/54 cases (19%).

Three cases (13%) had concomitance of class 5 and class 4 mutations; 6 cases (25%) had concomitance of class 5 and class 3 mutations and 15 (62%) had a single pathogenic mutation.

**Fig 3:**
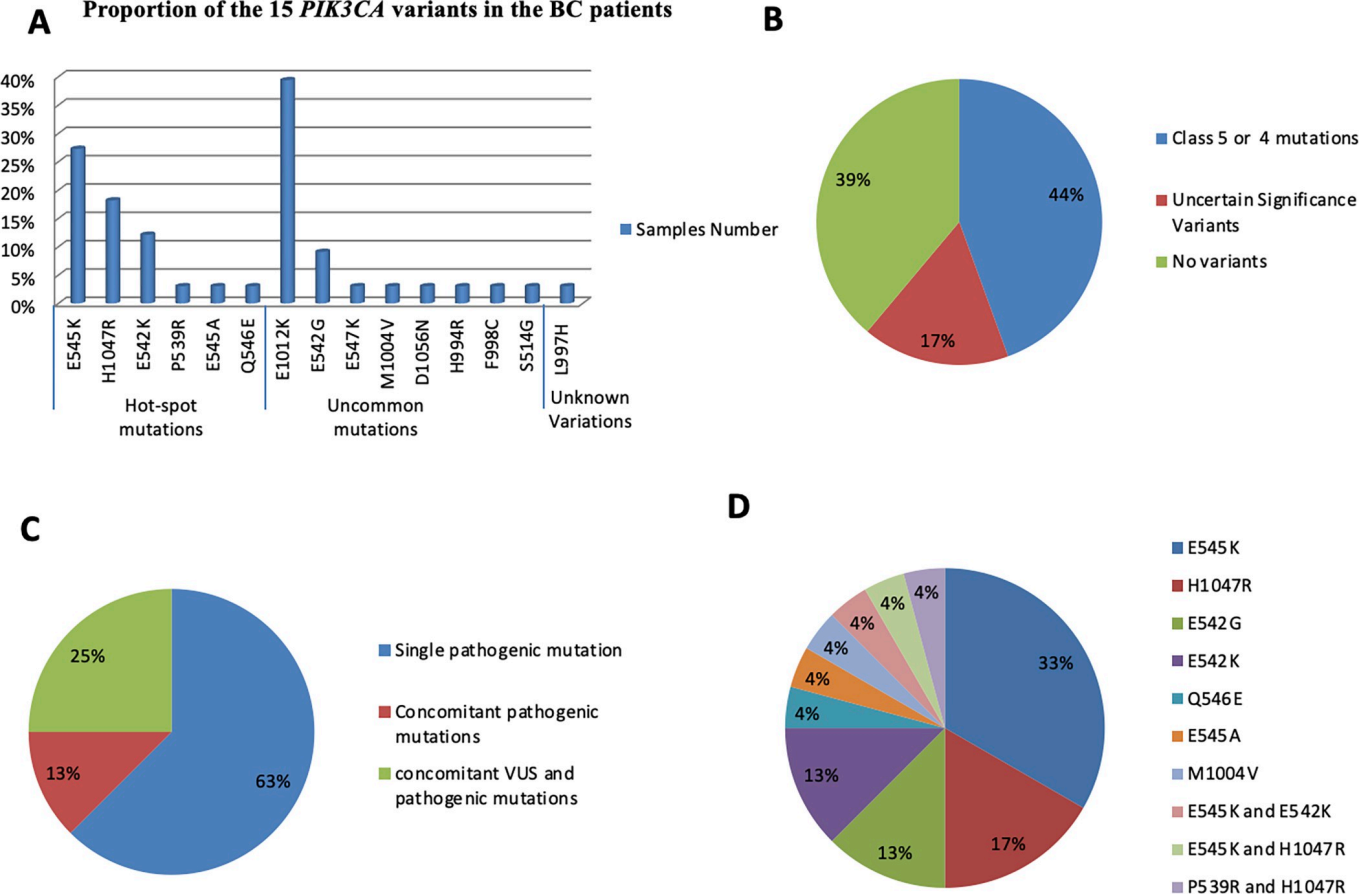
Distribution of mutation spectrum among breast cancer cases. (A) Frequencies of the fifteen identified *PIK3CA* variants among breast cancer samples. (B) Proportion of samples with class 5 and 4 mutations. (C) Proportion of samples with concomitant or single mutations. (D) Proportion of samples with pathogenic class 5 and 4 *PIK3CA* mutations.

Among the 15 identified *PIK3CA* variants, 7 were class 5 (tier I): 5 variants (p.P539R; p.E542K; p.E545K; p.H1047R; p.E545A) had clinical evidence and 2 (p.Q546E and p.E542G) had experimental evidence of *PIK3CA* gain of function. One variant (M1004V) was class 4 with likely *PIK3CA* gain of function. Five variants had been previously described in databases (H994R, F998C, D1056N, S514G and E1012K) with unknown clinical significance, and one variant L997H had never been previously described (Fig 2). The S514G had been described in a germinal state, and E1012K had been described in melanoma and lung cancers (Table 2). All the six variants were predicted to be pathogenic by at least one *In silico* analysis software. One variant (E547K) was likely neutral, classified class 2 with unknown function in therapeutic response (Figs 2 and 3 and Table 3).

**Table 2:** Summary of variants annotation using the reference sequence NM_006218.4 and classification according to ACMG.

Exon/ Domain	cDNA position	Protein position	Number of allele	ID number of Existing_variation	ClinVar Clinical_Significance	OncoKb description	TCGA, PanCancer Atlas	Drug response to PI3K pathway inhibitor	Described in other cancers in literature	ACMG Class
**Exon9** **Helical domain**	**c.1540A>G**	**p.S514G**	1	SCV002188925	Uncertain significance (previously described at germinal level)	Unknown	Unknown	Unknown	Cowden Syndrom [15,16]	3
	**c.1616C>G**	**p.P539R**	1	COSM759; COSV104381411; COSV55876380; VCV000376243.2; CA16602696; rs121913285	Likely pathogenic	Gain of Function [17]	Known	Sensitive [18]	Breast [19]and other cancers [20,21]	5
	**c.1624G>A**	**p.E542K**	4	COSM760; CM153078,COSV55873227,COSV55894248; VCV000031944.15; rs121913273; CA333572	Pathogenic, likely pathogenic	Gain of Function [22]	Known	Sensitive [15]	Breast [19]and other cancers [23]	5
	**c.1625A>G**	**p.E542G**	3	COSM761; COSV55876800; COSV55881194; COSV55893680; VCV000376475.3; CA16602909; rs1057519927	Likely pathogenic	Gain of Function Predicted [15]	Known	Sensitive [22]	Breast and other cancers [19]	5
	**c.1633G>A**	**p.E545K**	10	COSM763; CM126692; COSV55873239; COSV55878227; VCV000013655.25; CA123334; rs104886003	Pathogenic, likely pathogenic	Gain of Function [15]	Known	Sensitive [22]	Breast and other cancers [19]	5
	**c.1634A>C**	**p.E545A**	1	COSM12458; CM130273; COSV55873209; COSV55873220; COSV55892885; VCV000013659.6; CA123342; rs121913274	Likely pathogenic, pathogenic	Gain of Function [15]		Unknown	Breast and other cancers [19]	5
	**c.1636C>G**	**p.Q546E**	1	COSM6147; COSV55873527; COSV55882350 VCV000013654.1; CA123331; rs121913286	Pathogenic, likely pathogenic	Gain of function–predicted [15]		Unknown	Breast and other cancers [19]	5
	**c.1639G>A**	**p.E547K**	1	COSM29315; COSV55896899	Likely-neutral	Unknown	**Known**	Unknown	Breast [24]and other cancers	2
**Exon 20** **Kinase domain**	**c.2981A>G**	**p. H994R**	1	rs373295359	-	Unknown	Unknown	Unknown	Unknown	3
	**c.2990T>A**	**p.L997H**	1	Unknown	-	Unknown	Unknown	Unknown	Unknown	3
	**c.2993T>G**	**p. F998C**	1	COSV55943240	-	Unknown	Unknown	Unknown	Unknown	3
	**c.3010A>G**	**p.M1004V**	1	COSM7182160; COSV55914513	-	Unknown	Unknown	Unknown	Breast angiosarcoma and other cancers [25]	3
	**c.3034G>A**	**p.E1012K**	13	COSV55907590,COSV55914417	-	Unknown	Unknown	Unknown	Lung [26] ; Melanoma [27]	3
	**c.3140A>G**	**p.H1047R**	6	rs121913279,CM153086,COSV55873195,COSV55873401,COSV55888015	Pathogenic/ Likely-pathogenic	Gain of Function [15]	Known	Sensitive [28,29]	Breast and other cancers [19]	5
	**c.3166G>A**	**p. D1056N**	1	COSV55987728	-	Unknown	Unknown	Unknown	Unknown	3

**Table 3:** Prediction function and conservation of variants with unknown functions.

Exon/ Domain	cDNA position	Protein position	phyloP conservation (Score)	SIFT v6.2.0) (Score, median)	PolyPhen2 (Score)	Mutation Taster (v2021)
**Exon9**	**c.1540A>G**	**p.S514G**	Highly conserved (8.96)	Deleterious (0.00,: 4.32)	Possibly damaging (0.709); benign (0.187)	Benign
	**c.1639G>A**	**p.E547K**	Highly conserved (8.8)	Deleterious (0.01)	Probably damaging (0.964)	Deleterious
**Exon 20**	**c.2981A>G**	**p. H994R**	Highly conserved (8.85)	Deleterious (score: 0.05,3.33).	Possibly damaging (0.456)	Deleterious
**Kinase domain**	**c.2990T>A**	**p.L997H**	Highly conserved (7.60)	Deleterious (0.01, 3.33)	Probably damaging (1)	Deleterious
	**c.2993T>G**	**p. F998C**	Highly conserved (7.60)	Deleterious (0.01, 3.33)	Possibly damaging (0.863)	Deleterious
	**c.3010A>G**	**p.M1004V**	Highly conserved (8.82)	Tolerated (0.31, 3.33)	Benign (0.254)	
	**c.3034G>A**	**p.E1012K**	Highly conserved (9.24)	Tolerated (0.31, 3.33).	Probably damaging (0.999)	Benign
	**c.3166G>A**	**p. D1056N**	Highly conserved (9.37)	Tolerated (1.00, 3.33).	Possibly damaging (0.706)	Deleterious

A significant association was found between *PIK3CA* mutations and negative lymph node status (p = 0.027). No association was found between *PIK3CA* mutations and age, SBR grade, ER and PR status, HER2 overexpression, and molecular classification (Table 4).

**Table 4:** Association between PIK3CA mutations and clinicopathological characteristics of breast cancer.

Variables	Number of patients	PIK3CA mutations class 2 and 3 n (%)		PIK3CA mutations class 5 and 4 n (%)	p-value
**Median age**					0.394
<49	26	16 (62)	10 (38)		
≥49	28	14 (50)	14 (50)		
Total	54				
**SBR Grade**					0.227
I	4	2 (50)	2 (50)		
II	25	11 (44)	14 (56)		
III	25	17 (68)	8 (32)		
Total	54				
**Lymph node status**					**0.027**
Positive	29	19 (66)	10 (34)		
Negative	16	5 (31)	11 (69)		
Total	45				
**Estrogen receptors**					0.205
Positive	38	19 (50)	19 (50)		
Negative	16	11 (69)	5 (31)		
Total	54				
**Progesterone receptors**					0.577
Positive	36	21 (58)	15 (42)		
Negative	18	9 (50)	9 (50)		
Total	54				
**HER2**					0.201
Positive	4	1 (25)	3 (75)		
Negative	50	29 (58)	21 (42)		
Total	54				
**Molecular classification**					0.515
Luminal A	40	24 (60)	16 (40)		
Luminal B	5	2 (40)	3 (60)		
HER2+	4	1 (25)	3 (75)		
Triple negative	5	3 (60)	2 (40)		
Total	54				

## Discussion

In this study, we investigated the distribution of *PIK3CA* mutations in BCs of Tunisian women. We found a high frequency of *PIK3CA* mutations, particularly in exon 9. The *PIK3CA* mutated status was associated with negative lymph node status.

In our series, pathogenic and likely pathogenic *PIK3CA* mutations were identified in 44% of BCs. This rate is higher than that reported in The Cancer Genome Atlas (TCGA) (34.18%) [30]. The frequency of *PIK3CA* mutations varies according to the population studied and environmental factors. For example, it is reported in 29% of BCs in Japan [31] and in 34% of BCs in India [32]. It also varies with the number of exons analyzed and the techniques used. The highest rate of *PIK3CA* mutations detected by Sanger sequencing was 46.5% reported in a Chinese series using Sanger sequencing of 8 exons (1, 2, 4, 7, 9, 13, 18, and 20) of the *PIK3CA* gene [33].

Currently, targeted next generation sequencing (NGS) has become the most commonly used technique. It is recommended by ASCO for the detection of *PIK3CA* mutations for treatment eligibility for alpelisib among patients with luminal subtype BC [34]. Data concerning the concordance between Sanger sequencing and NGS are limited. Arsenic et al. [35] compared Sanger sequencing and NGS for the detection of *PIK3CA* hotspot mutations in exon 9 and exon 20 in 184 BCs and reported a concordance rate of 98.4%. NGS allows the identification of multiple mutations simultaneously, avoiding the need to perform sequential individual tests. Sanger sequencing is more expensive and labor-intensive [35,36]. However, it has the advantage of identifying novel variants. Our study identified six novel variants (H994R, L997H, F998C, D1056N, S514G, and E1012K) that have not been previously described in BCs. The E1012K variant has been described in lung cancer [26] and in melanoma [27] with an unknown functional effect.

In BCs, *PIK3CA* mutations are more commonly clustered in exon 20 than in exon 9, as reported in previous studies [26,28–30]. However, in our study, the prevalence of pathogenic *PIK3CA* mutations in exon 9 (17/24) was found to be higher than in exon 20 (5/24). Nevertheless, this result needs to be confirmed in larger series. The distribution of hotspot PIK3CA mutations in exon 9 and exon 20 accounted for 75% of class 5 and class 4 PIK3CA mutations in our series, which is similar to the rate reported in the literature [37,38]. The distribution of these PIK3CA hotspot mutations was found to be 55% for H1047R, 20% for E545K, and 11% for E542K [39].

Concomitant *PIK3CA* mutations are not uncommon in BCs. In our series, 13% of BCs had concomitant class 5 and class 4 PIK3CA mutations, and 25% had concomitance of class 5 and class 3 mutations. Lian et al. [40] reported one case of *PIK3CA* mutations in both exon 9 and exon 20 (E545K+H1047L) out of 43 mutated BCs. In a Chinese study, 17 of 537 (3.2%) BCs carried two mutations. Two of them had H1047R simultaneous with E542K or E545K [41]. Vasan et al. [42] demonstrated that the presence of double *PIK3CA* mutations on the same allele increases PI3K activity, which leads to enhanced downstream signal transduction, cell proliferation, and tumor growth. Concomitant mutations could be present in different tumor clones or may be present in the same tumor cell. Heterogeneity of *PIK3CA* mutational status has been previously described at the single cell level in circulating tumor cells from the same BC’s patient [43].

The association of *PIK3CA* mutations in BCs with clinicopathological features is controversial. In the present study, *PIK3CA* mutations were associated with negative nodal status but not associated with age, SBR grade, ER and PR status, HER2 overexpression and molecular classification. However, data from literature are conflicting. In some studies, *PIK3CA* mutations are associated with positive ER and PR status, negative HER2 expression [44–46], and negative nodal status [45,47,48]. The negative association between *PIK3CA* mutations and lymph node metastasis may be explained by the fact that actionable mutations in PIK3CA display constitutive activation of Akt [49]. Once Akt is activated, it promotes carcinogenesis in the early stages while suppressing tumor invasion and metastatic potential. It has been demonstrated that bitransgenic mice that express both activated Akt and ErbB2 in the mammary epithelium show increased breast tumor growth and a significant reduction in lung metastasis when compared to transgenic mice that express only activated ErbB2 [50]. Other reports found an association of *PIK3CA* mutations with positive lymph node status suggesting that activation of the PI3K/Akt pathway may increase the invasion of cancer cells into the lymph nodes [35,51–53].

## Conclusion

Our study shows a high frequency of *PIK3CA* mutations in BCs of Tunisian women, especially in exon 9. *PIK3CA* mutated status is associated with negative lymph node status. Further investigations should be undertaken in larger series exploring other exons and using more sensitive techniques such as NGS.
